# Bromide of Ammonia in Dysmenorrhœa

**Published:** 1872-05

**Authors:** 


Bromide of Ammonium in Dysmenorrhœa.—Dr. Hazelton (Proceedings Gynæcological Society, Gynæcological Journal, Feb., '72), in referring to a case of Dysmenorrhœa, said: "Cotton root, cinchona bark, etc., had been given without benefit. He then gave the patient ten grains of bromide of ammonium, repeating the same in two hours, with very great relief. He had perceived a similar effect in other cases, and was inclined to believe that the ammonium might be found to have the same effect in allaying uterine hyperæmia, as has the similar salt of potassium upon congestion of the base of the brain."

